# Cognition and psychomotor vigilance in treated sleep apnea patients with and without daytime sleepiness: the MAGNETO study

**DOI:** 10.1007/s44470-026-00077-9

**Published:** 2026-04-16

**Authors:** Barbara Junco, Alberto Ramos, Rene Hernandez-Cardenache, Douglas McKay Wallace, Salim Dib, Adriana P. Pérez Negrón, Alexandra Vanderkley, Roger McIntosh

**Affiliations:** 1 Department of Neurology, University of Miami, Miller School of Medicine, 5151 San Amaro Drive, Coral Gables, FL 33146 USA; 2 Department of Psychology, University of Miami, Coral Gables, FL USA; 3 Clinical and Translational Science Institute, University of Miami, Coral Gables, FL USA; 4 Department of Psychiatry, University of Miami, Miller School of Medicine, Coral Gables, FL USA; 5 Miller School of Medicine, University of Miami, Bascom Palmer Eye Institute, Coral Gables, FL USA

## Abstract

**Abstract:**

**Study Objectives:**

Residual excessive daytime sleepiness (EDS) persists in some patients with obstructive sleep apnea (OSA) despite adequate positive airway pressure (PAP) therapy and is linked to cognitive deficits. This study examined whether residual EDS in PAP-adherent OSA is associated with poorer cognition.

**Methods:**

A cross-sectional single site study (*N* = 65), examining PAP-adherent (mean use ≥ 6 h/night; device reported normalized AHI < 5 per hour) patients with OSA (mean age (SD) = 61 (9.2); 34% female; 72% Hispanic; 6% Black; mean diagnostic AHI = 38.05 (28.4). Objective sleepiness was measured via the Psychomotor Vigilance Task (PVT); > 5 lapses ≥ 500 ms defined PVT derived EDS (*N* = 30). Cognitive function assessed via NeuroTrax® battery accounting for age and education. General linear models adjusted for sex, time since diagnosis and self-reported sleep duration were used to test associations in this exploratory, hypothesis generating analysis.

**Results:**

Greater residual EDS was associated with worse performance in global cognition (β = -4.09, *p* < .001), memory (β = -3.97, *p* = .001), executive functioning (β = -3.91, *p* = .004), attention (β = -2.68, *p* = .036), and processing speed (β = -4.89, *p* = .012). In categorical analyses, those with EDS performed approximately .43-.58 of a NeuroTrax® SD worse in global cognition and memory, a magnitude roughly comparable to 1–2 points on the MoCA or MMSE.

**Conclusions:**

Residual EDS was associated with poorer cognitive performance. These findings suggest residual EDS represents a cognitive risk phenotype in OSA, independent of PAP adherence. PVT provides a brief, objective tool to detect cognitive deficits and may serve as a valuable measure in OSA related brain health research.

**Brief summary:**

**Current Knowledge/Study Rationale** In obstructive sleep apnea (OSA), residual excessive daytime sleepiness (EDS) can persist despite regular adherence to positive airway pressure (PAP) therapy. EDS is associated with cognitive decline and dementia risk, yet few studies have evaluated objective sleepiness and cognitive outcomes in this population.

**Study Impact** This study shows that residual EDS, objectively measured by the Psychomotor Vigilance Task (PVT), is associated with worse cognition in PAP-adherent people with OSA. These findings highlight a distinct cognitive risk phenotype in treated OSA and support the PVT as a practical tool for detecting cognitive deficits in clinical and research settings.

**Supplementary information:**

The online version contains supplementary material available at 10.1007/s44470-026-00077-9.

## Introduction

Obstructive sleep apnea (OSA) is a common and costly condition, affecting up to one billion people worldwide [1]. It results from repeated upper airway obstruction causing blockage of airflow while sleeping [2]. OSA produces intermittent hypoxemia, sleep fragmentation, and sympathetic activation [3], mechanisms associated with cognitive impairment [2] and chronic disease risk [4]. While positive airway pressure (PAP) therapy effectively reduces respiratory events and improves oxygenation, a subset of patients continues to experience residual daytime sleepiness despite PAP adherence [5–7].

Residual excessive daytime sleepiness (EDS) is a significant clinical concern, contributing to reduced quality of life, increased risk of accidents, and higher rates of mood, cardiovascular and neurocognitive comorbidities [6, 8, 9]. EDS is linked to persistent cognitive deficits, particularly in attention and processing speed, which are critical for higher order executive function and memory, and may involve OSA related structural and functional brain changes [10–12]. EDS in older adults has been consistently associated with an increased risk of dementia, even without confirmed OSA [12]. A meta-analysis of 18 longitudinal studies found that sleep disturbances, including EDS, were associated with a higher risk of all-cause dementia [13]. Similarly, in a 12-year study of French older adults, baseline EDS independently predicted all cause dementia, vascular dementia and faster global cognitive decline over 8 years [14, 15]. However, these studies primarily relied on self-reported symptoms of EDS, which can differ from objective measures of sleepiness.

On self-report instruments such as the Epworth Sleepiness Scale (ESS) [16], 22–34% of PAP- adherent patients with OSA endorse residual EDS [17], whereas objective tests like the Maintenance of Wakefulness Test (MWT) [18] and the Multiple Sleep Latency Test (MSLT) [19] suggest that up to 65% meet criteria [17]. This discrepancy implies that some patients may underreport or are unaware of their sleepiness. The cognitive consequences of residual sleepiness also remain understudied in PAP adherent OSA. Few studies have paired objective sleepiness testing with detailed cognitive assessment, highlighting the need for the present work. In PAP adherent OSA, a residual EDS phenotype is increasingly being recognized [5, 17], but identification is complicated by heterogeneity in subjective and objective measures. Prior neuroimaging studies in other cohorts suggest potential white matter alterations in these patients [20, 21]. Because attentional vigilance is among the first cognitive processes to worsen with inadequate sleep, we used the psychomotor vigilance task (PVT) as a reproducible, language independent metric of sustained attention and reaction time. The 10 min test is rapid, portable and clinic friendly compared to the day long MWT/MSLT protocols [22–24]. The PVT quantifies attentional lapses, mean reaction time, and response time variability, and is sensitive to both acute and chronic sleep loss [22, 24]. Performance also worsens with nocturnal hypoxemia in OSA, with studies associating oxygen desaturation to more lapses and slower response times [25]. Impaired PVT performance is associated with depression and mild cognitive impairment in several cognitive aging studies [26, 27]. These findings highlight the PVT’s value for detecting neurocognitive dysfunction in residual sleepiness that self-report may overlook.

This study examined PAP-adherent adults with OSA to determine whether residual EDS is associated with distinct patterns of cognitive dysfunction. We posited that persistent EDS may reflect downstream effects of OSA related physiologic stressors such as intermittent hypoxemia and sleep fragmentation, on neural systems supporting attention, processing speed, executive function and memory, consistent with ATS workshop frameworks [28–31]. Specifically, we expected patients with residual sleepiness to show poorer performance in global cognition, executive function, attention, verbal memory, and processing speed. Objective residual EDS, operationally defined by the PVT, was examined in relation to cognition as a hypothesis generating characterization of phenotype.

## Methods

### Population

A cross-sectional study was conducted to evaluate cognitive function and psychomotor vigilance in adults with OSA treated with PAP. The study followed STROBE guidelines for observational research and was approved by the Institutional Review Board at the University of Miami, Miller School of Medicine. Recruitment occurred at the Sleep Disorders Center at Bascom Palmer Eye Institute in Miami, FL and from a registry of individuals previously expressing interest in research studies. Recruitment began in December 2019, progressed slowly through April 2023 due to COVID-19 pandemic related disruptions, and was completed by July 2024. Community referrals were accepted if accompanied by valid in-laboratory polysomnography (PSG) records and reviewed for eligibility by the research team. Of 601 adults pre-screened, 304 were fully screened for eligibility. Of these, 145 were excluded, 65 enrolled, and 94 either declined participation or did not respond to contact attempts (see supplemental materials for participant flow diagram). All participants signed an informed consent form and were compensated for their time and effort. Participants over the age of 40 with a confirmed diagnosis of mild, moderate or severe OSA who had been using PAP therapy for at least six months were enrolled in the study. To meet eligibility criteria, participants were required to demonstrate consistent adherence, defined as an average use of six or more hours per night, and a normalized apnea–hypopnea index (AHI) (< 5 events per hour) based on data downloaded from their PAP devices. We excluded individuals with comorbid respiratory disease (e.g., COPD), prior stroke, coronary artery disease, heart failure, cardiac arrhythmias, or known carotid or intracranial arterial stenosis, as determined by medical history. Participants with central sleep apnea (central apnea index > 5 per hour and > 50% central events) or complex sleep apnea were excluded. Other exclusions included: current use of oxygen, periodic limb movement disorder with arousals (> 15 per hour), use of sedating medications, obesity hypoventilation syndrome, narcolepsy, dementia, other major neurological disorders (e.g., multiple sclerosis), neurodevelopmental disorders (e.g., ADHD), claustrophobia, or inability to communicate verbally.

All participants underwent either in-laboratory PSG or home sleep apnea testing as part of routine clinical care. Data collection included electroencephalography, electrooculography, electromyography, and detailed respiratory monitoring for those who completed in-lab PSG. Home sleep apnea tests (HSAT) provided limited channel data, capturing respiratory effort, airflow, and oxygen saturation but not sleep staging or arousal indices. In both modalities, participants with obstructive sleep apnea were defined by severity as having an apnea–hypopnea index (AHI) of 5–14.9 (mild), 15–29.9 (moderate) and ≥ 30 (severe) events per hour or respiratory event index [32, 33]. A certified sleep technologist manually scored each study, identifying apneas, hypopneas, oxygen desaturation events, and arousals. All studies were scored according to current American Academy of Sleep Medicine (AASM) guidelines [32, 33].

### Main outcome

Cognitive testing was performed in person under the supervision of a licensed neuropsychologist or trained neuropsychometrician. We used the NeuroTrax® computerized battery, a validated tool designed to assess multiple cognitive domains [34]. Cognitive domains and related assessments were as follows: Attention (Go-No Go, Stroop Interference, Staged Information Processing composite scores), Executive Function (Go-No Go, Stroop Interference, Catch Game composite), Information Processing Speed (Staged Information Processing composite), Memory (Verbal Memory, Delayed Verbal Memory, Non-Verbal Memory, Delayed Non-Verbal Memory composite), and Global Cognition (average of index scores). All scores were normalized for age and years of education.

### Exposures

Objective sleepiness was assessed using the PVT, a 10-min reaction time test administered during the research visit [28, 35]. The primary outcome was the number of lapses (reaction times ≥ 500 ms). Participants with > 5 lapses were categorized as EDS; those with ≤ 5 as no EDS, with cutoffs based on prior OSA studies using the PVT [20, 21].

#### Covariates and data collection

Demographic information (age, sex, race/ethnicity, income, education, and language preference) and medical history (time since OSA diagnosis, hypertension, diabetes, depression, anxiety, caffeine and alcohol intake, smoking status) and self-reported sleep duration were obtained via standardized questionnaires and review of the electronic medical record. Height and weight were collected at the time of testing to calculate body mass index (BMI). Obesity was defined as BMI ≥ 30 kg/m2. Subjective daytime sleepiness was captured with the Epworth Sleepiness Scale (ESS) at the study visit. A score > 10 was used to denote increased self-reported sleepiness [16]. Depression was measured via the Geriatric Depression Scale (GDS-15) [36] and anxiety was assessed via the Zung Self-Rating Anxiety Scale (SAS) [37] obtained as part of the NeuroTrax® battery. The majority of participants scored below the clinical threshold for anxiety and depression, thus these measures were not included as covariates in the primary analyses. The sample was also highly educated, with a mean of 16.8 years of education, which may influence cognitive performance outcomes. All assessments were available in English and Spanish and administered in the participant’s preferred language.

### Statistical analysis

All data were reviewed for quality and completeness, and any data entry errors were corrected following established quality control procedures. Missing data for predictors and outcomes were minimal, with one observation missing on the PVT and one on the cognitive outcomes. Given the low missingness regression analyses were conducted using complete case data. Descriptive statistics were calculated for all study variables, and some self report measures used only for descriptives had modest missingness (e.g., ESS 14%, SAS and GDS 17%). Box plots were used to identify extreme values, while histograms, skewness, kurtosis, the Shapiro Wilk test, and visual inspection were used to assess normality. Extreme outliers were winsorized at predefined percentile thresholds to reduce undue influence on statistical models. Variables that did not meet model assumptions were transformed prior to analysis, with the choice of transformation (e.g., square root, logarithmic) determined by each variable’s distribution and with the aim of improving residual normality.

Cognitive outcomes included global cognition, memory, executive function, attention, and information processing speed. Age and years of education were accounted for in the cognitive assessment norms, therefore were not included as covariates. Residual EDS was defined based on PVT performance and in line with prior literature [20, 21]. For descriptive statistics, participants were grouped categorically into EDS and no EDS. For linear regression models, residual EDS was analyzed continuously using the total number of lapses (≥ 500 ms), average reaction time (RT), and reaction time coefficient of variation (RTCV), defined as the within-task standard deviation of RT divided by the mean RT (SD/mean), a standard index of response-time variability. All regression models included assigned sex at birth, time since diagnosis and self-reported sleep duration as covariates.

For sensitivity analyses examining executive function, we additionally examined associations between each continuous PVT metric (lapses, RT, and RTCV) and the NeuroTrax executive function composite as well as individual executive subtests (Go–No–Go, Stroop Interference, and Catch Game). To evaluate potential departures from linearity, we fit restricted cubic spline models (4 degrees of freedom) for each PVT metric and compared spline-based and linear models using likelihood ratio tests.

We used generalized linear models (GLM) with Gaussian distribution and robust standard errors (HC3) to account for potential heteroskedasticity. The Benjamini–Hochberg false discovery rate (FDR) procedure was applied to adjust *p*-values for multiple comparisons across cognitive outcomes. Analyses were conducted using Python statistical software with significance set at *p* < 0.05 after FDR correction.

## Results

A total of 65 adults diagnosed with OSA, all of whom were adherent to PAP therapy, participated in the study. The mean age was 61 years, with 34% of the sample classified as female based on assigned sex at birth. Seventy-two percent of participants self-identified as Hispanic or Latino (Table 1). Forty-six percent met criteria for residual EDS based on PVT performance (i.e., > 5 lapses ≥ 500 ms). A substantial portion of the sample (48%) were classified as having severe OSA at initial diagnosis. Average nightly use of PAP therapy was 6.9 h (Table 1), and all participants met adherence criteria with normalized AHI.

**Table 1:** Descriptive statistics and group comparisons by excessive daytime sleepiness (EDS)

Variable	All (*n*=65)	EDS (*n*=30)	No EDS (*n*=35)	*p*-value
Demographics				
Age (years)	61.02 (9.24)	62.07 (8.90)	60.11 (9.55)	.400
Female (*n)*	22 (34%)	9 (30.0%)	13 (37.1%)	.544
Hispanic (*n)*	47 (72%)	21 (70.0%)	26 (74.3%)	.700
Black (*n)*	4 (6%)	3 (10.0%)	1 (2.9%)	.232
Education (years)	16.77 (2.93)	16.23 (3.08)	17.23 (2.75)	.173
*BMI (kg/m²)	29.50 (26.72 - 33.70)	30.25 (26.12 - 33.7)	28.8 (27.22 - 33.43)	.955
*AHI	26.35 (14.42 - 59.15)	50.1 (13.2 - 72.2)	24 (17.1 - 46.25)	.446
OSA severity at diagnosis				
Mild (AHI 5–14.9.9) (*n)*	17 (26.2%)	10 (34.5%)	7 (19.4%)	.223
Moderate (AHI 15–29.9.9) (*n)*	16 (24.6%)	3 (10.0%)	13 (37.1%)	.011
Severe (AHI ≥30) (*n)*	32 (49.2%)	17 (56.7%)	15 (42.9%)	.267
PAP therapy				
*PAP use (hours per night)	7 (6 - 7.5)	7 (6.5 - 7.5)	6.75 (6 - 7.5)	.126
Mood scales				
*SAS score	33.50 (28 - 43)	39.5 (32 - 43)	33 (27.75 - 39.25)	.190
*GDS-15 score	2 (1 - 4)	2 (1 - 5)	2 (1 - 3.25)	.363
Cognitive assessments				
Global cognition	101.3 (10.10)	97.87 (11.66)	104.08 (7.69)	.013
Executive function	105.3 (11.30)	103.03 (13.62)	107.09 (8.72)	.153
Memory	98.83 (11.02)	94.26 (12.68)	102.75 (7.58)	.001
Attention	99.81 (9.96)	98.03 (10.77)	101.28 (9.14)	.197
Info. processing speed	95.70 (20.74)	90.13 (20.59)	100.47 (19.92)	.044
Psychomotor Vigilance Test (PVT)				
*Reaction time (RT)	373.3 (335.4–427.8)	427.5 (380.0–508.2)	344.3 (313.0–368.5)	<.001
*RTCV	25.17 (19.07 - 33.34)	29.99 (24.46 - 46.75)	21.48 (16.90 - 26.99)	.002
*Total lapses (>500 ms)	5 (2 - 12.25)	14 (9 - 1)	2 (1 - 3.5)	<.001
*Number false starts	3 (1 - 8)	3 (1 - 8)	3 (1 - 7)	.903
*Number of errors	0 (0 - 2)	0 (0 - 2)	0 (0 - 2)	.870
Epworth Sleepiness Scale (ESS)				
*ESS total	6 (2 - 10)	5.5 (0.5 - 11.5)	7 (2 - 9)	1.00
ESS total > 10 (*n)*	12 (18.5%)	7 (23.3%)	5 (14.3%)	.351

*EDS* Excessive daytime sleepiness, *SD* Standard deviation, *RTCV* Reaction time coefficient of variation, *Min* Minimum, *Max* Maximum, *PAP* Positive airway pressure, *AHI* Apnea–hypopnea index, *SAS* Self-Rating Anxiety Scale, *GDS* Geriatric Depression Scale, *PVT* Psychomotor Vigilance Test. Between group comparisons performed using independent samples t-test, Mann–Whitney U test, or Chi-square test as appropriate. * Variables with skewed distributions are summarized using the median (25th, 75th percentiles)

When comparing subjective sleepiness between participants with and without EDS, results indicated that ESS scores between EDS groups were comparable (see Table 1). The correlation between ESS scores and PVT lapses was also small and non-significant (Spearman’s rho = 0.021, *p*= 0.876). Average ESS scores in both groups were below the conventional subjective EDS cut-off score of > 10. Most participants reported minimal mood symptoms. Based on established clinical cutoffs (SAS ≥ 45; GDS-15 ≥ 5), 85% scored below the threshold for clinically significant anxiety on the SAS, and 76% scored below the threshold for depression on the GDS-15 [36, 37].

Using robust GLM models adjusting for sex at birth, self-reported sleep duration, and time since OSA diagnosis, the association between objective measures of sleepiness (total lapses, average RT, and RTCV) and all cognitive outcomes were evaluated. A square root transformation was applied to all predictors to adjust for skewness.

Results indicated that greater total lapses were associated with lower performance in global cognition (standardized β = −4.09, *p* < 0.001), memory (β = −3.97, *p* = 0.001), executive functioning (β = −3.91, *p* = 0.004), attention (β = −2.68, *p* = 0.036), and slower information processing speed (β = −4.89, *p* = 0.012). These effects remained statistically significant after FDR correction for multiple comparisons. Similarly, longer average RT was associated with poorer global cognition (β = −3.50, *p* = 0.003), memory (β = −3.20, *p* = 0.009), executive functioning (β = −3.87, *p* = 0.002), and slower information processing speed (β = −4.09, *p* = 0.037), with all but attention (β = −2.44, *p* = 0.062) remaining significant after FDR adjustment (*p* < 0.05). In contrast, RTCV was not significantly associated with any cognitive outcome after FDR correction.

These findings indicate that objective indices reflecting the frequency and magnitude of attentional lapses (specifically total lapses and mean reaction time) were consistently associated with cognitive performance across multiple domains in PAP-adherent individuals with OSA, whereas variability in response speed was not independently associated with cognition after accounting for multiple comparisons (Table 2). Sensitivity analyses including additional adjustment for age, years of education, and BMI yielded similar results (Supplemental Table S1).

**Table 2:** Associations between psychomotor vigilance test (PVT) total lapses and average reaction time and cognitive function

	B	SE	z	*p*-value	95% CI Lower	95% CI Upper	β	FDR *p*-value
Total lapses*								
Global cognition	‒2.01	0.54	‒3.73	< 0.001	‒3.07	‒0.95	‒4.09	0.001
Memory	‒1.95	0.60	‒3.24	0.001	‒3.13	‒0.77	‒3.97	0.003
Exec. function	‒1.93	0.67	‒2.89	0.004	‒3.23	‒0.62	‒3.91	0.007
Attention	‒1.32	0.63	‒2.09	0.036	‒2.55	‒0.08	‒2.68	0.036
Info. Proc. Speed	‒2.41	0.96	‒2.51	0.012	‒4.29	‒0.52	‒4.89	0.015
Reaction time*								
Global cognition	‒1.17	0.39	‒3.02	0.003	‒1.93	‒0.41	‒3.50	0.006
Memory	‒1.07	0.41	‒2.63	0.009	‒1.87	‒0.27	‒3.20	0.041
Exec. function	‒1.30	0.42	‒3.06	0.002	‒2.13	‒0.47	‒3.87	0.006
Attention	‒0.82	0.44	‒1.87	0.062	‒1.67	0.04	‒2.44	0.062
Info. Proc. Speed	‒1.37	0.66	‒2.08	0.037	‒2.66	‒0.08	‒4.09	0.047
RTCV*								
Global cognition	‒0.38	0.86	‒0.44	0.657	‒2.07	1.30	‒0.74	0.738
Memory	‒1.47	0.72	‒2.05	0.041	‒2.87	‒0.06	‒2.84	0.203
Exec. function	‒1.06	0.73	‒1.45	0.146	‒2.49	0.37	‒2.05	0.366
Attention	0.17	0.51	0.33	0.738	‒0.83	1.18	0.33	0.738
Info. Proc. Speed	‒1.24	1.27	‒0.97	0.331	‒3.73	1.26	‒2.39	0.552

Each model adjusts for sex at birth, self-reported sleep duration, and time since OSA diagnosis. *Exec. Function.* Executive Functioning, *Info. Proc. Speed* Information Processing Speed, *B* unstandardized coefficient, *RTCV* Reaction time coefficient of variation, *SE* Standardized error, *CI* Confidence interval; β = Standardized coefficient, *FDR* False discovery rate correction. *p*-values < 0.05 are considered statistically significant. * PVT predictors were square rooted to adjust for skewness in data distribution

To aid clinical interpretation, unstandardized coefficients were expressed as a proportion of the NeuroTrax® standard score distribution (M = 100, SD = 15). For example, each one-unit increase in square-root–transformed total lapses was associated with reductions of 1.32 to 2.41 standard scores on cognitive indices, which corresponds to 0.09–0.16.09.16 of one standard deviation (SD = 15 points). Similarly, square-root–transformed average RT was associated with reductions of 0.82 to 1.37 standard scores, or 0.05–0.09.05.09 of a standard deviation.

Additional sensitivity analyses of executive function examined associations between PVT metrics (total lapses, average RT and RTCV) and individual executive function subtests. Results demonstrated a heterogeneous pattern wherein Catch Game showed significant associations with lapses and RT (both *p* < 0.01), and Stroop Interference was significantly associated with RT and RTCV (both *p* < 0.01). The Go-No-Go subtest showed no significant associations with any PVT metric (Supplemental Table S3). Further, likelihood ratio tests comparing spline based and linear models were not statistically significant for executive outcomes (all *p* > 0.05), indicating no evidence that nonlinear models improved fit.

We next evaluated cognitive differences between EDS groups using ANCOVAs, controlling for sex at birth, time since diagnosis, and self-reported sleep duration. Results indicated that participants with EDS (*M* = 97.87, *SD* = 11.66) performed significantly worse on global cognition than those without EDS (*M* = 104.28, *SD* = 7.71), *F*(1, 58) = 4.79, *p* = 0.033, partial eta squared (*η*_*p*_^*2*^) = 0.07. A similar pattern emerged for memory, with the EDS group (*M* = 94.26, *SD* = 12.68) performing significantly worse than the non-EDS group (*M* = 103.03, *SD* = 7.50), *F*(1, 49) = 10.43, *p* = 0.002, *η*_*p*_^*2*^ = 0.14. For clinical interpretation, the adjusted mean difference between groups corresponded to approximately 0.43–0.58.43.58 of a SD on the NeuroTrax® metric (global cognition: *M*_diff_ = 6.41 points; memory: *M*_diff_= 8.77 points). For context, widely used cognitive screens such as the MMSE and MoCA show standard deviations of approximately 2–3 points in large normative samples [38–40], meaning that a 0.5 SD difference on NeuroTrax is comparable in magnitude to a roughly 1–2-point shift on these instruments, which is clinically meaningful [41].

**Fig. 1:**
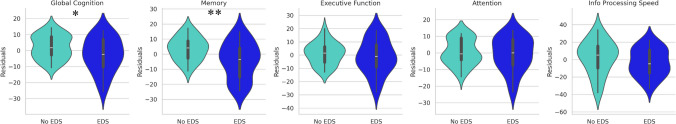
Violin plots of cognitive outcomes by excessive daytime sleepiness (EDS) status. Covariates included in adjustment include biological sex, sleep duration and time since OSA diagnosis. * *p* < 0.05, ** *p* < 0.01

Group differences were not statistically significant for executive functioning (EDS *M* = 103.0, *SD* = 13.82; no EDS *M* = 107.2, *SD* = 8.83; *F*(1, 58) = 0.98, *p* = 0.326, *η*_*p*_^*2*^ = 0.02), attention (EDS *M* = 98.03, *SD* = 10.77; no EDS *M* = 101.28, *SD* = 9.28; *F*(1, 58) = 0.32, *p* = 0.574, *η*_*p*_^*2*^ < 0.01), and processing speed (EDS *M* = 90.13, *SD* = 20.59; no EDS *M* = 100.74, *SD* = 20.16; *F*(1, 59) = 1.78, *p* = 0.188, *η*_*p*_^*2*^ = 0.02). The absence of significant group effects for these domains, despite significant associations in regression analyses, likely reflects reduced statistical power in the categorically defined EDS comparison relative to the continuous EDS predictor. See Fig. 1 for violin plots representing group differences in cognitive outcomes. Sensitivity analyses using alternative PVT total lapses thresholds are presented in Supplemental Table S2.

## Discussion

Residual EDS, operationalized using the PVT, was associated with poorer performance across all cognitive domains in optimally treated OSA. When viewed as a proportion of the NeuroTrax® SD, the magnitude of effects suggested clinically meaningful differences in cognitive performance. As this was a cross-sectional study, findings reflect associations rather than causal effects. Significant associations remained after adjusting for covariates and FDR correction. Total lapses and mean reaction time showed more consistent associations with cognition than reaction time variability.

Subjective sleepiness did not correlate with objective sleepiness, consistent with prior work showing limited agreement between subjective and objective measures of sleepiness [42, 43]. Jennum et al. [44] also reported that residual EDS persisted in 15.6% of PAP adherent patients despite follow up ESS in the normal range (< 10) [44]. ESS scores below the conventional cutoff should not be interpreted as an absence of functional impairment as objective lapses in vigilance may still be present. These findings highlight the clinical value of the PVT as a rapid, objective tool capable of detecting neurobehavioral impairment that self-report and laboratory measures like MWT/MSLT may miss [18, 19].

Prior residual EDS in treated OSA studies have reported the dominance of executive function deficits. In particular Werli et al. [45] observed selective impairments on executive tasks such as the Wisconsin Card Sorting Test (WCST) and semantic verbal fluency in patients with residual EDS, with relatively preserved memory and attention. The ATS workshop similarly highlighted executive and attention domains are most affected in OSA, while also suggesting significant heterogeneity across studies and outcomes [31]. Several methodological differences may explain why the present study shows broader associations across cognitive domains. Notably, the executive measures used by Werli et al. [45] focus primarily on executive control, strategy and rule shifting. The WCST and semantic verbal fluency tasks are not reaction time based. In contrast, the NeuroTrax executive function measures (e.g., Go-No-Go, Stroop Interference and Catch Game) rely on speed performance in which reaction time and temporal stability of response are central performance metrics. These tasks integrate executive control, sustained attention and processing speed, and may be particularly sensitive to vigilance deficits associated with sleepiness [24, 46]. Therefore, objective vigilance deficits as captured by the PVT may better reflect associations across executive, attention and processing speed related domains.

Cohort age further differentiates the present findings from those of Werli et al. [45]. In that study, both residual EDS and the control group had a mean age of approximately 51 years, whereas participants in the present study were on average nearly a decade older. Aging is associated with slowed processing speed and greater susceptibility to performance decrements on sustained attention and speeded tasks [47], which may contribute to broader domain involvement in older treated OSA cohorts. Cognitive outcomes may also vary as a function of age and task demands [31]. Accordingly, findings from younger cohorts such as Werli et al. [45] may not fully capture the broader cognitive impact of residual EDS observed in older PAP adherent OSA populations.

Participants in the present study were also highly educated. This may have attenuated categorical group differences through cognitive reserve mechanisms, while preserving sensitivity to continuous relationships between PVT defined EDS and cognitive outcomes. Additionally, cognitive reserve may further contribute to heterogeneity in cognitive outcomes among PAP adherent individuals.

Overall, continuous models found small to moderate associations between PVT captured EDS and cognitive outcomes across domains. However, categorical comparisons only showed significant group differences for global cognition and memory. This discrepancy is likely due to smaller effect sizes and loss of variance due to dichotomization, resulting in reduced sensitivity to detect effects and decreased statistical power. Specifically, many standardized mean differences fell within the small to moderate range (approximately 0.4–0.5 SD), which are detectable when EDS is modeled continuously but are more difficult to find in dichotomized group comparisons with modest sample sizes. Categorical analyses were therefore adequately powered to detect larger effect sizes, whereas smaller but meaningful effects likely resulted in nonsignificant findings. Given the sample size (*N* = 65), categorical analyses were likely powered to detect large (d ≥ 0.7) but not small to moderate (d≈0.4–0.5) effects. Additionally, confidence intervals around categorical group estimates were quite wide, suggesting limited precision in this sample and further restricting the ability to detect modest between group differences. This limitation is especially relevant for executive function subcomponents, which may show smaller effect sizes and greater within domain variability, increasing the likelihood of type II error in a sample of this size (*N* = 65). In this context, the pattern observed in the continuous analyses is consistent with a heterogeneous pattern of cognitive effects that likely vary by task demands.

Unlike prior studies focused on younger, non-Hispanic white males, our sample consisted of middle aged and older adults, the majority of whom were Hispanic-Latino, a population underrepresented in existing research on residual EDS in treated OSA. Later diagnosis and longer cumulative exposure to sleep disordered breathing have been reported in Hispanic-Latino populations [48]. This may contribute to more widespread cognitive effects, as sleep disordered breathing often goes undiagnosed and untreated in these groups [49]. Therefore, engaging and retaining diverse populations in sleep and cognition research is essential to characterizing variability in OSA related cognitive outcomes.

While many studies define EDS using the ESS or MSLT/MWT, this study used the PVT as the primary exposure. EDS was defined both continuously and categorically, using a threshold of > 5 lapses with reaction times ≥ 500 ms, consistent with prior research linking PVT defined sleepiness to impaired cognitive function [20, 21, 26, 50, 51].

Most work defines PAP adherence as ≥ 4 h per night, which may be insufficient to protect cognition, particularly in vulnerable populations [6, 52, 53]. Unlike studies relying on subjective sleepiness or showing limited cognitive improvement with wake promoting therapy, this study quantified residual EDS via the PVT which is language independent, objective, and a scalable measure of vigilance impairment, enabling detection of cognitive deficits. This approach may be useful in future clinical trials or observational studies examining cognitive consequences of residual EDS and its relevance to brain health in OSA [45, 53].

The ATS workshop [31] emphasizes dysregulation of vigilance and arousal systems, including frontoparietal control and thalamocortical arousal networks, as possible contributors to residual EDS [28–30]. Prior diffusion MRI studies of PAP-adherent patients with and without EDS have reported white matter microstructural differences, including increased radial diffusivity and reduced fractional anisotropy in patients with residual EDS [20, 21]. These changes correlate with the frequency of PVT lapses and persist even in PAP-adherent patients [20, 21]. Additionally, hippocampal vulnerability to intermittent hypoxemia may contribute to memory deficits [54–56]. In prior cohorts, hippocampal alterations have been observed in relation to subjective sleepiness, objective performance deficits, particularly in memory [54, 55, 57]. The hippocampus, sensitive to hypoxic injury, contributes to working memory and spatial navigation, functions impacted in EDS [54, 56]. Reduced hippocampal subfield volumes have been noted in OSA cohorts [58], supporting the possibility of medial temporal vulnerability within established frameworks of OSA related cognitive change [55]. Other studies link sleep fragmentation to disruption of frontal subcortical circuits supporting attention and executive control [11, 59].

The strengths of the study include the use of a standardized computerized cognitive battery administered alongside the PVT, all performed during a single research visit. There were strict inclusion and exclusion criteria applied, including objective verification of PAP adherence and exclusion of major neurological or psychiatric conditions, improving internal validity. Most participants scored in the normal range for anxiety and depression, indicating that these factors were unlikely to confound results. Our sample, comprised primarily of older Hispanic-Latino adults, represents a historically underrepresented population in sleep and cognition research, addressing a significant gap in the existing literature. While inclusion of this demographic represents a strength of this study, it also limits generalizability to other racial and ethnic groups. Differences in health profiles, access to treatment and sociodemographic factors across groups may influence the associations observed.

Limitations include the cross-sectional design, which prevents causal inference. Additionally, the use of the term “residual EDS phenotype” is intended as an operational and hypothesis generating description of a subgroup of PAP adherent individuals who display persistent EDS. PAP data were reviewed only for eligibility (mean nightly use and normalized AHI < 5). Other detailed device level metrics were not retained, which is a limitation given that residual physiologic burden may confound cognition even in adherent users [6]. While our sample size exceeds that of most prior studies on residual sleepiness, statistical power remains limited for detecting small effect sizes or conducting subgroup analyses. Participants underwent either polysomnography or type 3 home sleep apnea testing; the latter lacks EEG based sleep architecture and may underestimate respiratory event frequency. In addition, diagnostic metrics such as REM or supine related respiratory burden and detailed nocturnal hypoxemia indices were not available across participants, which prevents adjustment for these physiological factors. Nonetheless, it remains a widely accepted diagnostic tool for OSA. The high level of education in the sample may benefit cognitive reserve [60], possibly mitigating the magnitude of the observed cognitive impairments and limiting generalizability. Lastly, although our sample improves generalizability relative to prior work, findings may not extend to younger individuals or to females, who were underrepresented in this cohort.

## Supplementary information

Below is the link to the electronic supplementary material.

ESM 1 (DOCX 18.3 KB)

ESM 2 (DOCX 15.3 KB)

ESM 3 (DOCX 21.1 KB)

ESM 4 (PNG 311 KB)

## Data Availability

The University of Miami Human Subjects Research Office requires the establishment of a Data Use Agreement (DUA) for limited data sets containing protected health information. Data cannot be shared publicly because they contain protected health information. Data may be available with restrictions (contact: bxj107@med.miami.edu) for researchers who meet the criteria for access to confidential data.
