# Supplementary material for: Cognition and psychomotor vigilance in treated sleep apnea patients with and without daytime sleepiness: the MAGNETO study
Source: J Clin Sleep Med. 2026 Apr 16;22(1):60. doi: 10.1007/s44470-026-00077-9 (PMC13087004; doi:10.1007/s44470-026-00077-9)
Supplement: Supplementary file 1 — (DOCX 18.3 KB) [file 44470_2026_77_MOESM1_ESM.docx]

**Supplemental Table S1.** *Results of Sensitivity Analyses Investigating Associations Between Psychomotor Vigilance Test (PVT) and Cognitive Outcomes*

| **Model 1** | | |  |  | **Model 2** | |  |
| --- | --- | --- | --- | --- | --- | --- | --- |
| **Total Lapses*** | **B(SE)** | **β** | ***p*-value** |  | **B(SE)** | **β** | ***p*-value** |
| Global Cognition | -2.01(0.54) | -4.09 | 0.000 |  | -2.09(0.56) | -4.33 | 0.000 |
| Memory | -1.95 (0.60) | -3.97 | 0.001 |  | -1.92(0.62) | -3.97 | 0.002 |
| Executive Functioning | -1.93(0.67) | -3.91 | 0.004 |  | -1.90(0.69) | -3.94 | 0.006 |
| Attention | -1.32(0.63) | -2.68 | 0.036 |  | -1.29(0.64) | -2.67 | 0.043 |
| Info. Proc. Speed | -2.41(0.96) | -4.89 | 0.012 |  | -2.45(0.96) | -5.08 | 0.010 |
| **Reaction Time*** |  |  |  |  |  |  |  |
| Global Cognition | -1.17(0.39) | -3.50 | 0.003 |  | -1.29(0.40) | -3.90 | 0.001 |
| Memory | -1.07(0.41) | -3.20 | 0.009 |  | -1.04(0.43) | -3.14 | 0.015 |
| Executive Functioning | -1.30(0.42) | -3.87 | 0.002 |  | -1.32(0.47) | -3.99 | 0.005 |
| Attention | -0.82(0.44) | -2.44 | 0.062 |  | -0.80(0.46) | -2.42 | 0.085 |
| Info. Proc. Speed | -1.37(0.66) | -4.09 | 0.037 |  | -1.04(0.59) | -4.07 | 0.022 |
| **RTCV*** |  |  |  |  |  |  |  |
| Global Cognition | -0.38(0.86) | -0.74 | 0.657 |  | -0.41(0.89) | -0.82 | 0.643 |
| Memory | -1.47(0.72) | -2.84 | 0.041 |  | -1.53(0.71) | -3.04 | 0.031 |
| Executive Functioning | -1.06(0.73) | -2.05 | 0.146 |  | -1.02(0.76) | -2.03 | 0.180 |
| Attention | 0.17(0.51) | 0.33 | 0.738 |  | 0.33(0.53) | 0.65 | 0.530 |
| Info. Proc. Speed | -1.24(1.27) | -2.39 | 0.331 |  | -1.49(1.13) | -2.96 | 0.188 |

**Note.** Model 1 represents regression coefficients for the effect of PVT lapses controlling sex at birth, time since diagnosis, and self-reported sleep duration. Model 2 represents Model 1 + controls for age, education, and BMI. B = unstandardized coefficient; RTCV = reaction time coefficient of variation; SE = standardized error; β = standardized coefficient; *p*-values <.05 are considered statistically significant. * PVT predictors were square rooted to adjust for skewness in data distribution.
