# Supplementary material for: Cognition and psychomotor vigilance in treated sleep apnea patients with and without daytime sleepiness: the MAGNETO study
Source: J Clin Sleep Med. 2026 Apr 16;22(1):60. doi: 10.1007/s44470-026-00077-9 (PMC13087004; doi:10.1007/s44470-026-00077-9)
Supplement: Supplementary file 2 — (DOCX 15.3 KB) [file 44470_2026_77_MOESM2_ESM.docx]

**Supplemental Table S2.** Sensitivity Analyses Investigating Excessive Daytime Sleepiness (EDS) Group Differences in Cognitive Outcomes Based on Different Psychomotor Vigilance Task Total Lapses Cut-offs.

| **Global Cognition** | | | **Memory** | | **Executive Functioning** | | **Attention** | | **Info. Proc.**  **Speed** | |
| --- | --- | --- | --- | --- | --- | --- | --- | --- | --- | --- |
| **Cut-off** | ***ηp²*** | ***p*-value** | ***ηp²*** | ***p*-value** | ***ηp²*** | ***p*-value** | ***ηp²*** | ***p*-value** | ***ηp²*** | ***p*-value** |
| >3 | .02 | 0.21 | .04 | 0.102 | .01 | 0.348 | <.01 | 0.717 | .01 | 0.286 |
| >5 | .07 | 0.033 | .14 | 0.002 | .02 | 0.326 | <.01 | 0.574 | .02 | 0.188 |
| >7 | .04 | 0.092 | .09 | 0.013 | .01 | 0.481 | .02 | 0.296 | <.01 | 0.640 |
| >10 | .05 | 0.079 | .10 | 0.011 | .03 | 0.158 | .03 | 0.183 | .01 | 0.414 |
| >15 | .06 | 0.039 | .04 | 0.097 | .04 | 0.116 | .02 | 0.196 | <.01 | 0.566 |

Note. *ηp²* = Partial eta squared; Info. Proc. Speed = Information Processing Speed
