# Supplementary material for: Cognition and psychomotor vigilance in treated sleep apnea patients with and without daytime sleepiness: the MAGNETO study
Source: J Clin Sleep Med. 2026 Apr 16;22(1):60. doi: 10.1007/s44470-026-00077-9 (PMC13087004; doi:10.1007/s44470-026-00077-9)
Supplement: Supplementary file 3 — (DOCX 21.1 KB) [file 44470_2026_77_MOESM3_ESM.docx]

**Supplemental Table S3.** *Executive Function sensitivity analysis*

| **A.** | |  |  |  |  |  | **B.** |  |  |
| --- | --- | --- | --- | --- | --- | --- | --- | --- | --- |
| PVT Predictor | | Executive Function Outcome | B(SE) | *p*-value | β |  | χ²(df=3) | *p*-value |  |
|  |  |  |  |  |  |  |  |  |  |
| Total  Lapses | | EF Composite | -1.93(0.67) | 0.004 | -3.91 |  | 6.90 | 0.075 |  |
|  | | Go-No-Go | -0.74(0.88) | 0.403 | -1.50 |  | 4.10 | 0.250 |  |
|  | | Stroop Interference | -1.49(0.92) | 0.106 | -3.03 |  | 7.06 | 0.070 |  |
|  | | Catch Game | -1.85(0.67) | 0.006 | -3.75 |  | 2.67 | 0.445 |  |
| Reaction Time | | EF Composite | -1.3(0.42) | 0.002 | -3.87 |  | 2.05 | 0.562 |  |
|  | | Go-No-Go | -0.7(0.64) | 0.274 | -2.09 |  | 2.59 | 0.460 |  |
|  | | Stroop Interference | -1.76(0.47) | 0.000 | -5.25 |  | 0.79 | 0.851 |  |
|  | | Catch Game | -1.44(0.4) | 0.000 | -4.29 |  | 1.59 | 0.661 |  |
| RTCV | | EF Composite | -1.06(0.73) | 0.146 | -2.05 |  | 5.72 | 0.126 |  |
|  | | Go-No-Go | -0.54(0.89) | 0.546 | -1.05 |  | 4.46 | 0.216 |  |
|  | | Stroop Interference | -2.78(0.88) | 0.002 | -5.41 |  | 3.57 | 0.312 |  |
|  | | Catch Game | -1.73(1.1) | 0.115 | -3.37 |  | 6.40 | 0.094 |  |
|  |  | | | | | | | |  |
|  | | **C.** | EDS | No EDS | *t*-test | | | |  |
|  | |  | *M*(*SD*) | *M*(*SD*) | *t* |  | *p*-value | *g* |  |
|  | | EF Composite | 103.03(13.62) | 107.09(8.72) | 1.45 |  | 0.153 | 0.36 |  |
|  | | Go-No-Go | 99.32(17.57) | 98.65(14.57) | -0.17 |  | 0.867 | -0.04 |  |
|  | | Stroop Interference | 105.88(15.8) | 111.07(13.08) | 1.43 |  | 0.159 | 0.36 |  |
|  | | Catch Game | 108.08(13.42) | 111.28(10.32) | 1.09 |  | 0.282 | 0.27 |  |
|  | | **D.** |  |  | ANCOVA | | | |  |
|  | |  | *EMM*(*SE*) | *EMM*(*SE*) | *F* |  | *p*-value | *η²_p_* |  |
|  | | EF Composite | 103.8(2.15) | 106.6(1.92) | 0.98 |  | 0.326 | 0.38 |  |
|  | | Go-No-Go | 99.94(3.01) | 97.29(2.69) | 0.45 |  | 0.507 | 0.01 |  |
|  | | Stroop Interference | 105.6(99.74) | 111.7(2.64) | 2.37 |  | 0.129 | 0.04 |  |
|  | | Catch Game | 108.2(2.32) | 110.7(210) | 0.64 |  | 0.426 | 0.01 |  |

Note. (A) Linear regression models examining associations between continuous Psychomotor Vigilance Task (PVT) metrics and executive function outcomes, adjusted for sex at birth, time since obstructive sleep apnea (OSA) diagnosis, and self-reported sleep duration. (B) Non-linear associations evaluated using restricted cubic spline models (4 degrees of freedom); χ² statistics and p-values correspond to likelihood ratio tests comparing spline-based models with linear models. (C) Independent-samples t-tests comparing participants with and without excessive daytime sleepiness (EDS); values shown are unadjusted means with standard deviations (SD). Standardized mean differences are reported as Hedges g. (D) Analysis of covariance (ANCOVA) models comparing EDS and no-EDS groups, adjusted for sex at birth, time since diagnosis, and self-reported sleep duration; values shown are estimated marginal means (EMM) with standard errors (SE). Effect sizes for ANCOVA models are reported as partial eta squared (*η²_p_*). PVT metrics include total lapses (reaction times ≥500 ms), mean reaction time (RT), and reaction time coefficient of variation (RTCV; SD/mean). Executive function outcomes include the NeuroTrax® executive function (EF) composite and individual subtests (Go–No–Go, Stroop Interference, and Catch Game). Regression coefficients are reported as unstandardized B(SE), with β denoting standardized coefficients. Positive Hedges g values indicate higher cognitive performance in the no-EDS group.
