# Supplementary figures and images for: Cognition and psychomotor vigilance in treated sleep apnea patients with and without daytime sleepiness: the MAGNETO study
Source: J Clin Sleep Med. 2026 Apr 16;22(1):60. doi: 10.1007/s44470-026-00077-9 (PMC13087004; doi:10.1007/s44470-026-00077-9)

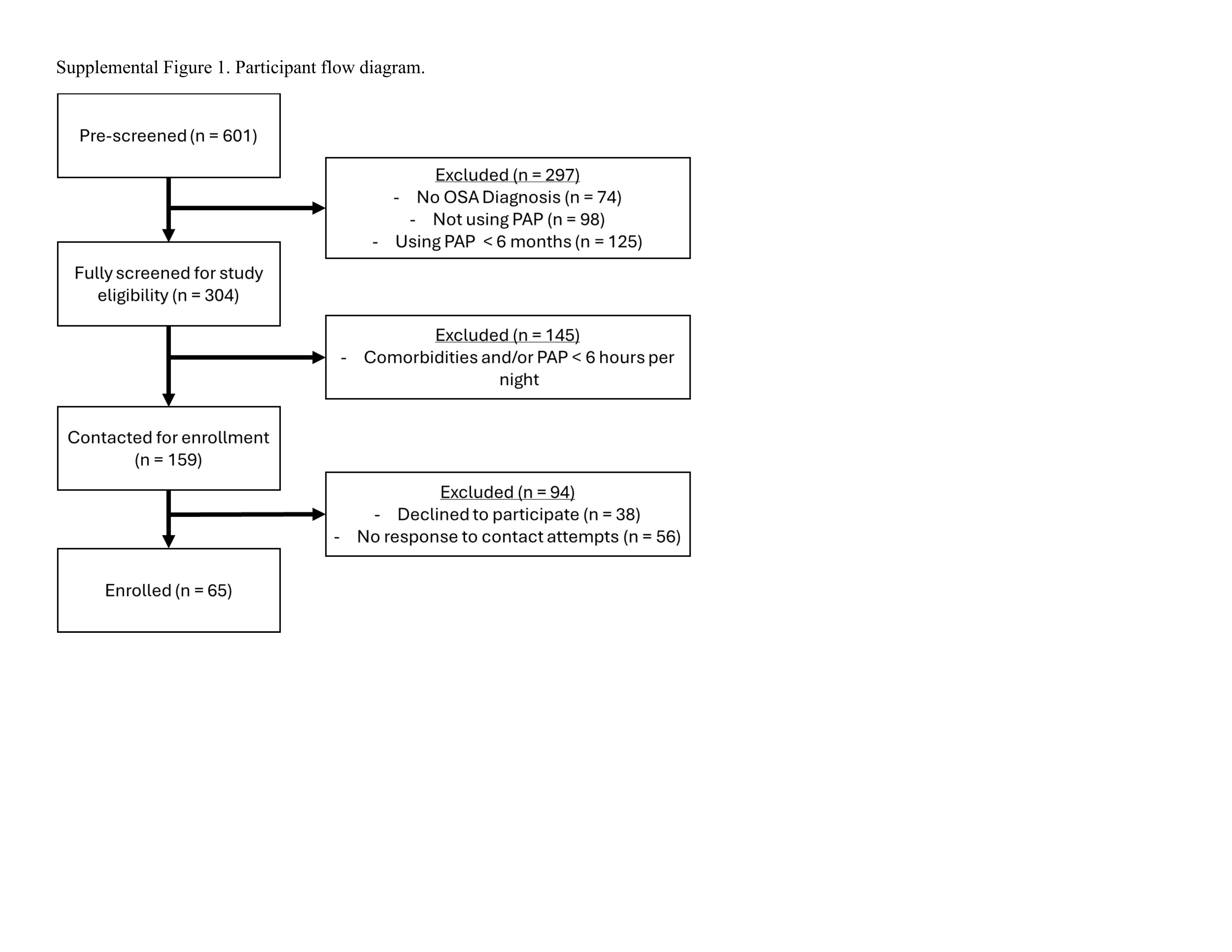

Supplement: Supplementary file 4 — (PNG 311 KB) [file 44470_2026_77_MOESM4_ESM.png]
